# Symptomatic Abdominal Wall Schwannoma Mimicking Infected Subcutanous Soft Tissue Lesion. A Case report

**DOI:** 10.1016/j.ijscr.2021.105751

**Published:** 2021-03-11

**Authors:** Abdullah G. Alsahwan, Jomana M. Felemban, Anas Al-Othman, Shahad Y. Assiri, Ali A. Alzahir

**Affiliations:** Department of General Surgery, Surgical Oncology Section, King Fahad Specialist Hospital-Dammam, Saudi Arabia

**Keywords:** Schwannoma, Soft tissue, Benign, Lumps

## Abstract

**Introduction and importance:**

Schwannomas are benign tumors of the nerve sheath, they do not typically present on the abdominal wall and cause symptoms. To the best of our knowledge, this is the second symptomatic case of schwannoma in upper abdominal wall reported according to the reviewed English medical literature.

**Case presentation:**

A 25-year-old man post renal transplant two year ago. He presented with left hypochondrial painfull swelling, and redness. On examination, local examination revealed a lesion located in the anterior abdominal wall swelling, 3 × 3 cm, tender with minimal erythema and hotness but no discharge. An ultrasound showed superficial oval shaped with thick wall and mildly increased peripheral vascularity without internal vascularity. He underwent surgical excision and the histopathology result was schwannoma.

**Clinical discussion:**

Usually, a patient presents asymptomatic with a slow growing mass. However, the symptoms can vary and depending on the location. Furthermore, a growing lump can start putting pressure on the nerves around the area, and patients can show symptoms accordingly. The likability of ultrasound to pick up a well-circumscribed mass and reaching a definitive diagnosis without histopathology is nearly impossible. Therefore, a complete excision and histopathology confirmed the presence of schwannoma.

**Conclusion:**

Upon the rarity of schwannomas presenting in atypical regions, such as the abdominal wall. A painful mass on the abdominal wall should raise the suspicion of benign schwannoma. The recurrence rate after the treatment of choice is unusual. Moreover, complete surgical excision of the mass is the definitive treatment.

## Highlights

• Schwannomas are benign tumors of the nerve sheath, these tumors can present anywhere but mostly in the extremities, trunk, head and neck but can present in rare locations such as the abdominal wall.
• Definitive diagnosis of the schwannomas without histopathology is nearly impossible and remain the cornerstone for diagnosis.
• Complete surgical excision is the ultimate treatment of choice for these lesions.

## 1 Introduction

Tumors of the Schwann cells that are found in the peripheral nerve sheaths are benign tumors that are called schwannoma [1]. These tumors can present anywhere but mostly in the extremities, trunk, head and neck, and usually found incidentally [2]. However, they can present in unusual locations such as the abdominal wall, as subcutaneous lesions and surgical excision is the definitive treatment [3].

## 2 Case report

A 25-year-old man post renal transplant two year ago. He presented to the emergency department with left hypochondrial swelling, that he noticed three months ago. It became painful for 2 weeks duration with no changes in the size or discharge from the lesion. He denied a history of trauma and fever. He is non-smoker, his psychosocial, drug and family history were unremarkable.

On examination, the patient was vitally stable, looks well and not in pain. Abdominal examination revealed a lesion located in the anterior abdominal wall, the area between the left hypochondrial and lower chest. The swelling was about 3 × 3 cm, tender with minimal erythema and hotness but no discharge.

**Fig. 1:**
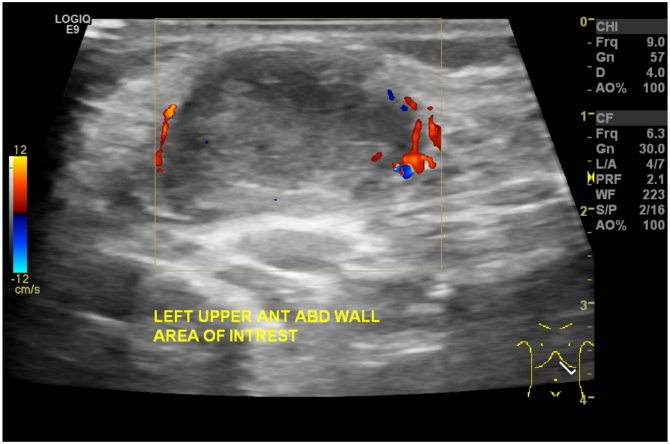
B-mode ultrasound using linear probe showing anterior abdominal wall 3.2 cm rounded well encapsulated heterogeneous predominantly hypoechoic mass with peripheral vascularity is located underneath the skin and displacing the surrounding fat planes.

The routine laboratory works up were unremarkable. An ultrasound was done while scanning the left anterior abdominal wall at the area of concern, it showed superficial oval shaped with thick wall and mildly increased peripheral vascularity without internal vascularity [Fig. 1]. In correlation to the clinical information of tenderness and redness, the findings are more likely going with infected subcutaneous soft tissue lesion, managed with oral antibiotic.

He then presented to the clinic a week later with the same history given previously, a surgical excision proposed to the patient and was done under local anesthesia by general surgeon. During the procedure, the excised mass was found to be round in shape, soft tissue in character, adhesion to the surrounding tissue which was excised with healthy margins and sent for histopathology review.

**Fig. 2:**
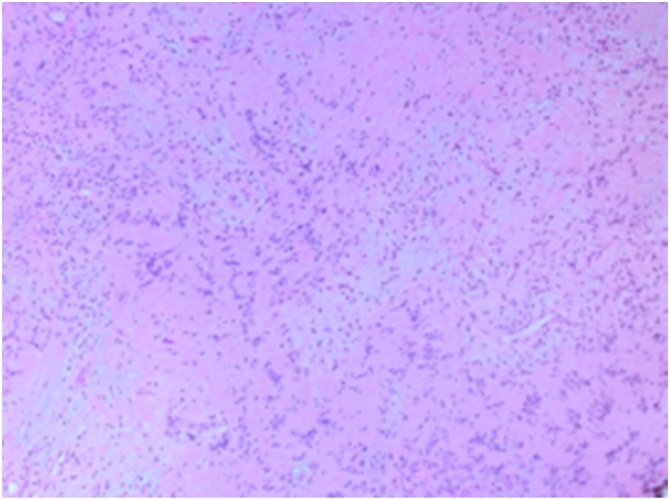
H&Ex10: Spindle cell tumor with prominent nuclear pallisading (Verocay Bodies).

**Fig. 3:**
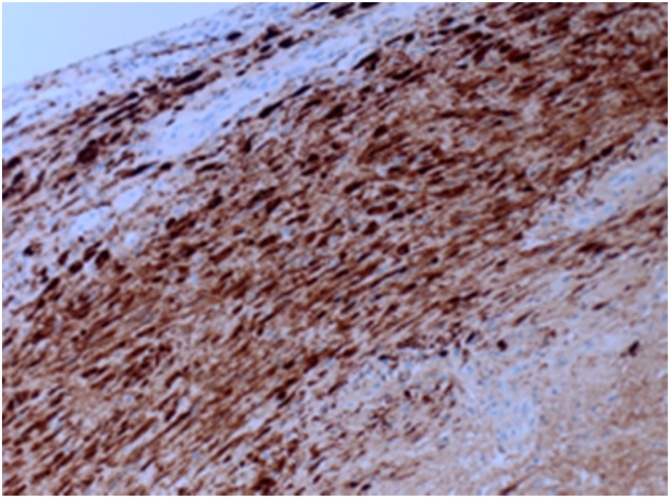
H&Ex20: Intralesional histiocytes and hyalinized blood vessels noted (Ancient changes).

**Fig. 4:**
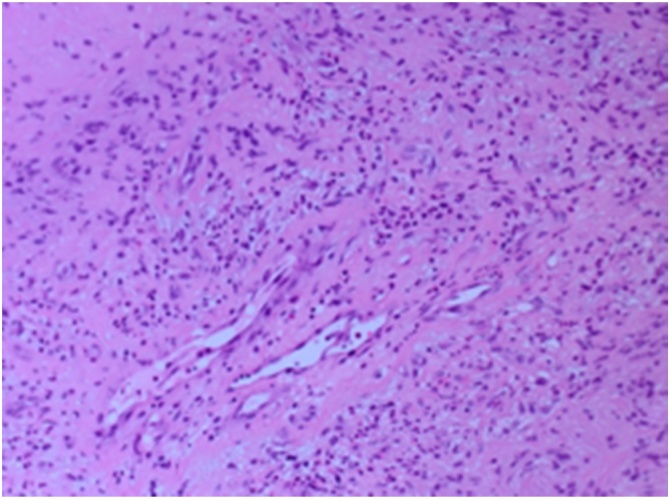
S100+: Diffuse and strong staining for S100 protein.

The histopathology findings of the lesion show a necrotic tissue with peripheral spindle cells with prominent nuclear pallisading (Verocay Bodies) [Fig. 2]. The spindle cells have indistinct cell border, eosinophilic cytoplasm. and spindle to plump nuclei. Well controlled immunostains show that the tumor cells are positive for S100 [Fig. 3], but negative for CD34, SMA, Desmin, beta-catenin, c-KIT and DOG-1. The final diagnosis was schwannoma with ancient changes i.e. (Intralesional histiocytes and hyalinized blood vessels) [Fig. 4] and negative for malignancy.

The patient had uneventful procedure with no complications and was discharged home in a good condition and followed regularly in the clinic, He experienced improvement in his symptoms, quality of life and the capability of coping with daily demands.

## 3 Discussion

Schwan cells of the peripheral nerve sheath is the origin of the benign neurogenic tumors, they are usually encapsulated and slow growing, they can present with pain, paresthesia, or less commonly with neurologic deficits [1,6]. Transformation of benign schwannoma to malignancy is rare, nevertheless malignant schwannomas exist and they are about 5–10% of sarcomas [5,7].

As often known, schwannomas do not affect the conductivity of the nerve that arises from it. Usually, a patient presents asymptomatic with a slow growing mass. However, the symptoms can vary and depending on the location they present differently [1]. Furthermore, a growing lump can start putting pressure on the nerves around the area, and patients can show symptoms accordingly.

Although imaging of superficial subcutaneous lesions is usually ultrasound [3], computed tomography (CT) or magnetic resonance imaging (MRI) can be used. Where, MRI is the most reliable of 61% sensitivity and 90% specificity [1,3]. The appearance of schwannoma on CT or MRI as a well-defined homogeneous mass [1,2,5].

To aid the diagnosis of these lesions, histopathology is strongly recommended. Typically, schwannomas react strongly with S100 protein and immunohistochemistry [5,7]. Where they help differentiating benign schwannomas from other malignant tumors by the presence of hyperchromatic cells and nuclear atypia [1,6,7].

Histologically, schwannoma is a well encapsulated mass, composed of typical dense cellular areas (Antoni type A) and myxoid areas (Antoni type B) [1,4] Ancient schwannoma as diagnosed in our patient, is often named after long-standing schwannomas. Where, a phenomenon of degenerative changes throughout the time and atypia is suggestive of ancient schwannomas [3,4].

## 4 Conclusions

Upon the rarity of schwannomas presenting in atypical regions, such as the abdominal wall. A painful mass on the abdominal wall should raise the suspicion of benign schwannoma [5]. The recurrence rate after the treatment of choice is unusual [4,6]. Moreover, complete surgical excision of the mass is the definitive treatment [[3], [4], [5], [6],^8^].

## Declaration of Competing Interest

The authors report no declarations of interest.

## Sources of funding

This study did not receive any funding from governmental or private organizations.

## Ethical approval

Ethical approval was obtained from the Institutional Review Board (IRB) of the King Fahad Specialist Hospital, Dammam, Saudi Arabia. The ethical approval was signed on 01^st^ February 2021.

## Registration of research studies

Not applicable.

## Author contribution

Abdullah G. Alsahwan, Jomana M. Felemban, Anas Al-Othman, Shahad Y. Assiri and Ali A. Alzahir: study concept or design, data collection, data interpretation, literature review, drafting of the paper, final review of the manuscript.

## Guarantor

Dr. Abdullah Ghazi AlSahwan.

## Provenance and peer review

Not commissioned, externally peer-review.
